# Co-localization of IgG with nephrin in immune-mediated idiopathic nephrotic syndrome

**DOI:** 10.1007/s10157-025-02741-5

**Published:** 2025-08-06

**Authors:** Yuta Ichikawa, Nana Sakakibara, Shuhei Aoyama, Yuka Kimura, Yuta Inoki, Yu Tanaka, Chika Ueda, Hideaki Kitakado, China Nagano, Tomohiko Yamamura, Shingo Ishimori, Yuko Shima, Hayaki Okamoto, Hideki Fujii, Hironobu Maruyama, Kazumoto Iijima, Kandai Nozu, Tomoko Horinouchi

**Affiliations:** 1 Department of Pediatrics, Kobe University Graduate School of Medicine, 7-5-1 Kusunoki-cho, Chuo, Kobe, Hyogo 6500017 Japan; 2 Department of Pediatrics, Wakayama Medical University, Wakayama City, Japan; 3 Division of Nephrology and Kidney Center, Kobe University Graduate School of Medicine, Kobe, Japan; 4 Immuno-Biological Laboratories, Fujioka, Japan; 5 Hyogo Prefectural Kobe Children’s Hospital, Kobe, Japan; 6 Department of Advanced Pediatric Medicine, Kobe University Graduate School of Medicine, Kobe, Japan

**Keywords:** Nephrin, Anti-nephrin antibodies, Idiopathic nephrotic syndrome, Nephrin/IgG co-localization, Nephrin/IgG cocktail antibodies

## Abstract

**Background:**

Increased serum anti-nephrin antibody titers and co-localization of nephrin and IgG in kidney tissues have been reported in minimal change disease (MCD) and post-transplant recurrent focal segmental glomerulosclerosis (FSGS). These results indicate an association of anti-nephrin antibodies with nephrotic syndrome (NS); however, the exact relationship remains unclear. Herein, we evaluated nephrin/IgG co-localization in the glomeruli of patients with various kidney diseases, including monogenic NS, to clarify the association between idiopathic nephrotic syndrome (INS) and anti-nephrin antibodies.

**Methods:**

IgG and nephrin co-localization was investigated in 52 kidney tissue biopsy samples, comprising INS in the active phase (*n* = 26; MCD, *n* = 19; FSGS, *n* = 7) and remission (*n* = 6), monogenic NS (*n* = 3), and other kidney diseases (*n* = 17). Double-immunofluorescence staining for nephrin/IgG was performed in unfixed frozen sections for 2 h at room temperature with Alexa Fluor-labeled nephrin/IgG cocktail antibodies. Nephrin/IgG co-localization was assessed using optical sectioning under a fluorescence microscope.

**Results:**

Nephrin/IgG co-localization was observed in 81% (21/26, children: 15/17, adults: 6/9) of active INS cases, 84% (16/19) of MCD cases, and 71% (5/7) of FSGS cases. No co-localization was observed in NS with monogenic variants or other kidney diseases.

**Conclusion:**

Nephrin/IgG co-localization in the kidney tissue is finding observed in active INS**,** strongly indicating an association between anti-nephrin antibodies and INS onset. The nephrin/IgG cocktail antibody is a rapid and effective approach for investigating INS pathogenesis that facilitates the differential diagnosis of immune-mediated NS from other kidney diseases, including monogenic NS.

**Supplementary Information:**

The online version contains supplementary material available at 10.1007/s10157-025-02741-5.

## Introduction

Nephrotic syndrome is an umbrella term for glomerular filtration-barrier dysfunction, which results in generalized edema because of heavy proteinuria and hypoalbuminemia, due to various factors, such as immune abnormalities, infections, and genetic background. The glomerular filtration barrier is composed of multiple structural and functional proteins, among which nephrin, a slit-diaphragm-related protein of podocytes, is one of the most important components [1, 2]. Idiopathic nephrotic syndrome (INS), especially minimal change disease (MCD), is the most common glomerular disease, particularly in children. INS, which develops through immunological mechanisms, has long been thought to be caused by immune cell dysregulation and the production of circulating factors that impair the glomerular filtration barrier [3–5]. Nevertheless, the detailed pathophysiology has long been unclear [6]. However, in 2022, circulating nephrin autoantibodies (anti-nephrin antibodies) were identified in about 30% of a subset of nephrotic syndrome patients in acute phase with MCD pathology. The study also reported that punctate IgG was present in a subset of MCD glomeruli, co-localized with nephrin, which was considered to be anti-nephrin antibody [7]. Thereafter, several reports have strongly supported that anti-nephrin antibody is one of the etiologic factors of nephrotic syndrome [8–10]. A more recent study clarified that an elevation of the serum anti-nephrin antibody levels was seen in about 80% of INS cases [9]. Another study reported that plasma levels of anti-nephrin antibodies in post-transplant recurrences of focal segmental glomerulosclerosis (FSGS) were high during the recurrence period, and that graft biopsies at relapse showed punctate IgG deposits co-localized with nephrin, and that these punctate IgG disappeared after remission [10].

These data suggested that nephrin/IgG co-localization might be a finding observed in patients with immune-mediated INS. However, no comprehensive studies of nephrin/IgG co-localization in the glomeruli of patients with glomerular disease have been reported to date, and whether this co-localization is specific to INS has not been investigated. If nephrin/IgG co-localization is found to be specific to INS, it would be clinically useful and have high potential as a diagnostic tool, as distinguishing INS from other kidney diseases is often necessary. This distinction is particularly challenging in case of steroid-resistant nephrotic syndrome, where differentiating between immune-mediated INS and monogenic nephrotic syndrome based on clinical and pathological findings alone is difficult, while the treatment strategy is completely different [11]. A rapid distinction based on a kidney biopsy would be clinically valuable.

In this study, we evaluated nephrin/IgG co-localization in the glomeruli of patients with various kidney diseases, including nephrotic syndrome caused by monogenic factors, to clarify the association between the development of INS and anti-nephrin antibodies.

## Materials and methods

### Patients

In this study, we enrolled patients who underwent kidney biopsy at our institution and at affiliated hospitals between 2017 and 2024, for whom frozen tissue of the kidney biopsy, a detailed clinical course, and treatment information were available.

**Fig. 1:**
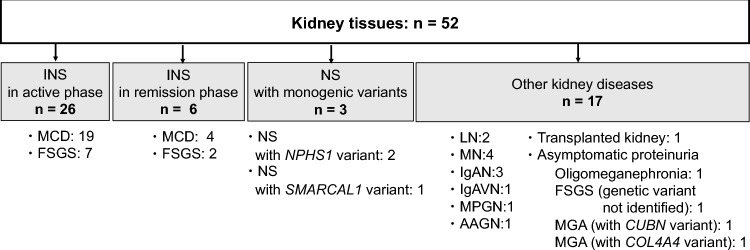
The list of kidney diseases examined in this study. This study included 52 kidney tissue samples, comprising INS in the active phase (*n* = 26), INS in remission (*n* = 6), NS with monogenic variants (*n* = 3), and other kidney diseases (*n* = 17), obtained between February 2017 and February 2024. *INS* idiopathic nephrotic syndrome; *MCD* minimal change disease; *FSGS* focal segmental glomerulosclerosis; *NS* nephrotic syndrome; *LN* lupus nephritis; *MN* membranous nephropathy; *IgAN* IgA nephropathy; *IgAVN* IgA vasculitis nephritis; *MPGN* membranoproliferative glomerulonephritis; *AAGN* ANCA-associated glomerulonephritis; *MGA* minor glomerular abnormalities

Fifty-two kidney tissue samples with various diseases were evaluated (Fig. 1). For clinically diagnosed INS, 32 samples obtained from children and adults were included, consisting of two pathological types: MCD (including those with mesangial proliferation) and FSGS. In pediatric patients with first-onset INS, routine kidney biopsies are not generally obtained. Reasons for kidney biopsies in this study are listed in Table S1. Of these 32 samples, 26 were collected during periods of high disease activity, characterized by apparent proteinuria (active phase), while six samples were collected during periods when proteinuria was absent (remission phase). Paired (active and remission) samples from the same patients were available for three individuals. Additionally, three samples of nephrotic syndrome associated with monogenic variants and 17 samples of various other kidney diseases were enrolled as control samples. Other kidney diseases included lupus nephritis (*n* = 2), membranous nephropathy (*n* = 4), IgA nephropathy (*n* = 3), IgA vasculitis nephritis (*n* = 1), membranoproliferative glomerulonephritis (*n* = 1), ANCA-associated vasculitis (*n* = 1), transplanted kidney (original disease: hypo-/dysplastic kidney) (*n* = 1), asymptomatic proteinuria due to oligomeganephronia (*n* = 1), asymptomatic proteinuria with FSGS (genetic variant not identified; *n* = 1), asymptomatic proteinuria with minor glomerular abnormalities (MGA) due to a *CUBN* variant (*n* = 1), and asymptomatic proteinuria with MGA due to a *COL4A4* variant (*n* = 1). The detailed information of these patients is shown in Table S1.

### Genetic analysis

A comprehensive analysis of podocyte-related genes was performed in 15 patients, as previously reported [12, 13]. The details of patients who underwent genetic testing are shown in Table S1.

The analyzed genes are listed in Table S2. Samples for next-generation sequencing were prepared using HaloPlex or SureSelect target enrichment system kits (Agilent Technologies, Santa Clara, CA, USA), in accordance with the manufacturer’s instructions. All indexed DNA samples were amplified using polymerase chain reaction and were sequenced on the MiSeq platform (Illumina, San Diego, CA, USA). Data for the alignment to the mutation categorization processes were analyzed using SureCall software (Agilent Technologies).

The pathogenicity of the detected variants was assessed according to the criteria and guidelines of the American College of Medical Genetics and Genomics [14]. The genotype details of the five cases in which disease-causing variants were identified are shown in Table S3.

### Double-immunofluorescence staining of nephrin and IgG

For rapid and convenient evaluation of the co-localization of nephrin and IgG in the frozen kidney sections, we prepared a premixed antibody (1 vial dual color cocktail antibody) of Alexa 546-labeled anti-nephrin antibody and Alexa 488-labeled anti-IgG antibody.

An Alexa 546-labeled anti-nephrin antibody was produced by incubating Alexa Fluor™ 546 NHS Ester (A20002; Invitrogen, Carlsbad, CA, USA) with anti-human nephrin (N) rabbit polyclonal IgG (Cat. No. 29050) (Immuno-Biological Laboratories, Fujioka, Japan) and purified using an antigen-specific column and its specificity is supported by the observation that it shows negative staining in kidney tissues from patients with *NPHS1* null variants, where nephrin protein is absent. An Alexa 488-labeled IgG antibody was produced by incubating the Alexa Fluor™ 488 NHS Ester (A20100; Invitrogen, Carlsbad, CA, USA) with anti-human IgG (11A3) mouse monoclonal IgG, affinity purified (not commercially available; Immuno-Biological Laboratories, Fujioka, Japan), which was produced using standard hybridoma technology, and purified from hybridoma culture supernatant using a Protein A column. The specificity of this antibody was confirmed by enzyme immunoassay (EIA), in which HRP-conjugated 11A3 antibody showed no cross-reactivity with human IgM or IgA, while confirming reactivity with all human IgG subclasses (IgG1, IgG2, IgG3, and IgG4). Unbound Alexa Fluor was then removed by gel filtration using P4Bio-Gel (Bio-Rad Laboratories, Hercules, CA, USA). These two antibodies were mixed in a ratio of 1:1.

Double-immunofluorescence staining of nephrin and IgG was performed using frozen sections prepared from kidney biopsy specimens. Frozen Sections (3 μm) were washed with 10% phosphate-buffered saline (PBS). Without fixation and blocking, sections were incubated with the nephrin/IgG cocktail antibody (1:50) at room temperature for 2 h. After three washes in 10% PBS, sections were mounted using Vectashield antifade mounting medium with DAPI (Vectashield; H-1200, Vector Laboratories, Newark, CA, USA) and were then viewed under a fluorescence microscope (BZ-X810; KEYENCE, Osaka, Japan) equipped with the optical sectioning algorithm system to obtain double fluorescence staining images. The obtained images were analyzed with image analysis software BZ-X800 (KEYENCE).

Since nephrin was stained red and IgG was stained green, areas where IgG and nephrin overlapped appeared yellow, which was defined as positive nephrin/IgG co-localization.

## Results

A summary of immunostaining results for nephrin/IgG co-localization is shown in Table 1. Among the 26 INS patients in the active phase, 21 (81%) were positive for co-localization of IgG and nephrin, whereas all three patients with monogenic nephrotic syndrome and all 17 patients with other kidney diseases were negative for co-localization. Furthermore, only 17% (1 of 6) of specimens in the remission phase were positive for co-localization.

**Table 1:** Numbers of patients with nephrin/IgG co-localization-positive disease

Disease	Number of cases (*n*)	Nephrin/IgG co-localization (+)
**INS in the active phase**	**26**	**21**
Children	17	15
Adults	9	6
MCD	19	16
FSGS	7	5
**INS in remission**	**6**	**1**
MCD	4	1
FSGS	2	0
**NS with monogenic variants**	**3**	**0**
NS with *NPHS1* variant	2	0
NS with *SMARCAL1* variant	1	0
**Other kidney diseases**	**17**	**0**
LN	2	0
MN	4	0
IgAN	3	0
IgAVN	1	0
MPGN	1	0
AAGN	1	0
Transplanted kidney	1	0
Asymptomatic proteinuria Oligomeganephronia	1	0
Asymptomatic proteinuria FSGS (genetic variant not identified)	1	0
Asymptomatic proteinuria MGA (with *CUBN* variant)	1	0
Asymptomatic proteinuria MGA (with *COL4A4* variant)	1	0

*INS* idiopathic nephrotic syndrome; *MCD* minimal change disease; *FSGS* focal segmental glomerulosclerosis; *NS* nephrotic syndrome; *LN* lupus nephritis; *MN* membranous nephropathy; *Ig* immunoglobulin; *IgAN* IgA nephropathy; *IgAVN* IgA vasculitis nephritis; *MPGN* membranoproliferative glomerulonephritis; *AAGN* ANCA-associated glomerulonephritis; *MGA* minor glomerular abnormalities

**Fig. 2:**
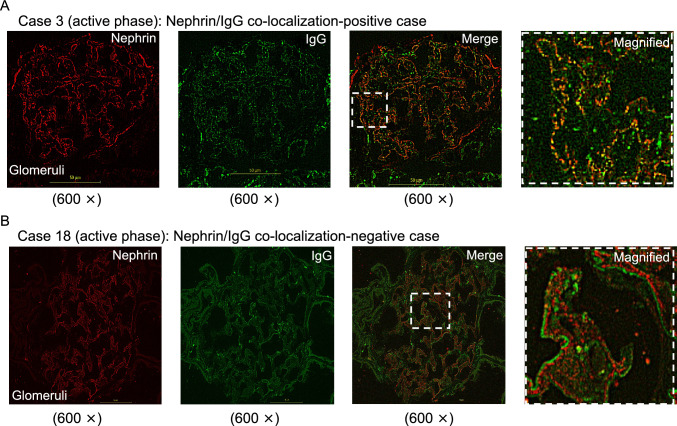
Representative double-immunofluorescence staining for nephrin and IgG. Double-immunofluorescence staining of frozen sections of biopsies obtained from patients with idiopathic nephrotic syndrome. First column, nephrin staining; second column, IgG staining; third column, merged image; fourth column, magnified image of the boxed area in the merged image. Nephrin in the glomeruli is labeled red, and IgG in the glomeruli is labeled green. When these images are overlaid, nephrin and IgG co-localization appear yellow. **A** Case 3 (active phase) shows a co-localization-positive pattern. When the image is magnified, the red nephrin and the green IgG clearly overlap, and the co-localization appears yellow. **B** Case 18 (active phase) shows a co-localization-negative pattern. In magnified image, the red nephrin and the green IgG regions clearly do not overlap

Figure 2A shows a patient with nephrotic syndrome in the active phase (Case 3) who exhibited co-localization of nephrin and IgG. In contrast, Fig. 2B shows another patient with nephrotic syndrome in the active phase (Case 18) who tested positive for IgG, but no yellow stain, indicating co-localization, was not observed. The staining results for all cases are shown in Fig. S1. Nephrin/IgG co-localization was generally observed as puncta along the glomerular capillary wall. However, in Case 20 and Case 25, IgG and nephrin overlapped and appeared yellow, but the pattern could not be described as punctate. Furthermore, in some cases, nephrin-bound IgG immune complexes were observed within the epithelial cells of the proximal tubules (Fig. S2).

**Fig. 3:**
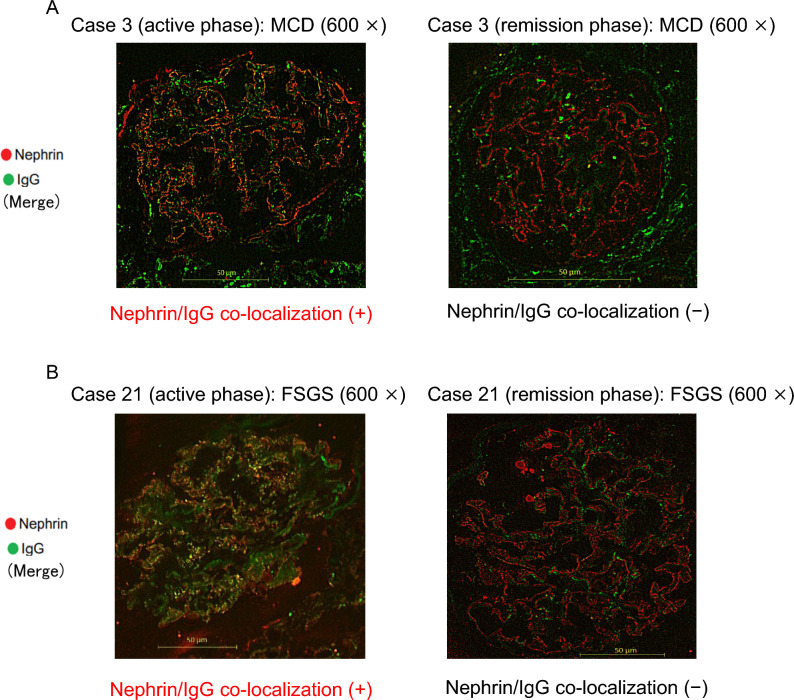
Comparison of nephrin/IgG co-localization between active and remission phases in the same idiopathic nephrotic syndrome case. Merged images of nephrin and IgG staining in Case 3 (**A**) and Case 21 (**B**) in both active phase and remission phase. The pathological findings on light microscopy were MCD in Case 3 and FSGS in Case 21. Nephrin and IgG colocalize in the active phase (left columns). However, nephrin and IgG no longer colocalize during remission (right columns). *MCD* minimal change disease; *FSGS* focal segmental glomerulosclerosis

In the three cases in which the active and remission phases could be evaluated in pairs, the two co-localization-positive specimens from patients in the active phase were co-localization-negative during the remission phase (Fig. 3), and the case that was negative in the active phase remained negative in the remission phase.

When divided by the time of onset of INS, nephrin/IgG co-localization was observed in 88% (15 of 17) of pediatric-onset cases (age at onset: 0–16 years) and in 67% (6 of 9) of adult-onset cases (age at onset: 47–88 years). Based on histopathology, nephrin/IgG co-localization was present in 16 of 19 patients (84%) with MCD and five of seven patients (71%) with FSGS.

Nephrin staining in patients with nephrotic syndrome, especially during the acute phase, tended to appear fainter and more punctate compared to other glomerular diseases. Even among patients in the acute phase, partial recovery of the linear nephrin staining pattern was observed in some pediatric MCD cases (cases 2, 3, and 14) who had received prednisolone for 4 weeks. In remission-phase cases, nephrin staining tended to appear more intense and linear compared with that in the acute-phase cases.

## Discussion

In this study, after comprehensive examination of glomerular kidney disease, we revealed that nephrin/IgG co-localization in kidney tissue is a finding observed in active-phase INS, regardless of its histopathological findings. Nephrin/IgG co-localization was generally observed along the glomerular capillary wall in INS, with most cases exhibiting a punctate pattern.

In kidney tissues other than INS, IgG and nephrin were clearly present in different locations. In some non-INS cases, IgG and nephrin were observed along closely spaced parallel lines (Fig. S3A), or IgG and nephrin were seen alternately in stripes (Fig. S3B, C), but never overlapped. In lupus nephritis, in which various autoantibodies are produced, punctate IgG was clearly observed, but no nephrin/IgG co-localization whatsoever occurred. This might mean that antibodies against podocyte proteins other than nephrin could play a pathological role in lupus nephritis. Similarly, in membranous nephropathy, although substantial IgG deposition occurred and despite its association with nephrotic syndrome, co-localization of nephrin/IgG was not observed.

In this study, nephrin/IgG co-localization was not observed in remission phase of INS, except in 1 case. In 2 cases where co-localization was positive during the active phase, it became negative in the remission phase, which is consistent with the previous serum analysis results [7–9]. In 1 case (Case 29), nephrin/IgG co-localization was observed even in complete remission, indicating that anti-nephrin antibodies may remain in kidney tissues despite clinically complete remission. Although co-localization was indeed positive in Case 29, the degree was mild, and the linear structure of nephrin was relatively preserved. Notably, in some cases, nephrin-bound IgG immune complexes were detected in the epithelial cells of the proximal tubules, following the structure of the proximal tubular epithelial cells. For example, in Case 21, which was in the active INS, co-localization was observed in both glomeruli and proximal tubules. Interestingly, in Case 27, which was a case of INS in remission, the co-localization at the glomeruli had already disappeared, but remained present in the proximal tubules (Fig. S2). Furthermore, Case 19 showed positive co-localization during the subclinical phase. In this case, proteinuria was detected by chance urinalysis, and at the timing of the kidney biopsy, the patient had not yet developed hypoalbuminemia in the nephrotic phase, and was in a subclinical phase with only asymptomatic proteinuria. However, the patient developed hypoalbuminemia soon thereafter, was diagnosed with nephrotic syndrome, and achieved complete remission with corticosteroid monotherapy. However, it remains unclear whether anti-nephrin antibodies become positive in kidney tissue before urinary protein appears.

Furthermore, co-localization was negative in all three cases of monogenic nephrotic syndrome and in two cases of asymptomatic proteinuria associated with monogenic variants (cases 30, 31, 32, 48, and 49). Notably, the specificity of the anti-nephrin antibodies used in this study was also demonstrated through staining in cases of monogenic nephrotic syndrome: a congenital nephrotic syndrome patient with compound heterozygous *NPHS1* variants (Case 31) showed no expression of nephrin in the glomeruli (Fig. S4), while patients with mild podocyte damage, such as nephrotic syndrome in remission or asymptomatic proteinuria, showed clear expression of nephrin. Thus, evaluating nephrin/IgG co-localization, which indicates the presence of anti-nephrin antibodies, is likely to be useful in differentiating monogenic nephrotic syndrome from immune-mediated INS. Although serum anti-nephrin antibodies have not been evaluated in nephrotic syndrome with monogenic variant, our study revealed that nephrotic syndrome with monogenic variant was negative for nephrin/IgG co-localization in kidney tissue. This may suggest that anti-nephrin antibodies are a cause rather than a consequence of nephrotic syndrome.

In this study, we encountered several cases where nephrin/IgG co-localization was not observed, despite a definitive diagnosis of INS. These cases developed rapidly and achieved complete remission after administration of some immunosuppressants. In these co-localization-negative INS, a clear deposition of IgG occurred, which sometimes overlapped the basement membrane (Case 18 and Case 23) or was surrounded by nephrin (Case 12) (Fig. S3D–F). These observed IgG depositions might represent some unidentified autoantibodies other than those against nephrin. For example, in mice, it has been reported that autoantibodies against CRB2, a slit-diaphragm-associated protein, cause podocyte injury, resulting in nephrotic syndrome [15]. Furthermore, anti-slit diaphragm antibodies other than anti-nephrin antibodies have also been identified in patients with steroid-resistant nephrotic syndrome　who respond to immunosuppressive therapy [16]. However, for IgG staining that does not colocalize with nephrin, it remains unclear whether this represents meaningful deposition targeting other antigens or nonspecific binding. Similar findings have been reported in the previous studies [7, 10], including IgG staining along Bowman’s capsule, which may be influenced by the use of highly sensitive antibodies or detection methods. These observations highlight the need for cautious interpretation of IgG signals that are not clearly associated with nephrin.

The INS group in this study included patients whose light microscopic findings were consistent with either FSGS or MCD. In this study, we identified anti-nephrin antibody-positive cases among both MCD and FSGS patients. The presence of anti-nephrin antibody-positive cases in both MCD and FSGS has also been reported previously [7, 9, 10, 17]. Although MCD and FSGS are classified as distinct pathological entities, we speculate that they may share a common underlying mechanism of disease onset. Differences in disease severity may account for the divergent histological presentations. At present, it remains unclear why similar pathogenic mechanisms lead to different pathological features; however, factors such as variations in serum titers of anti-nephrin antibodies or differences in IgG subclasses may be involved and warrant further investigation.

The study has some limitations. First, we were unable to assess circulating anti-nephrin antibody, because we conducted our study using archived kidney biopsy samples. An analysis of the relationship between circulating anti-nephrin antibody titer and glomerular nephrin/IgG co-localization intensity should be conducted in future. Second, we have employed optical sectioning with KEYENCE microscopy, while the previous studies have used confocal microscopy or stimulated emission depletion microscopy to determine nephrin/IgG co-localization in kidney tissue [7, 10, 16]. It is considered highly advantageous for routine clinical use due to its simplicity and its ability to achieve functionality equivalent to sectioning in confocal microscopy. However, the assessment of whether overlapping signals truly colocalize may not be sufficient. Particularly for patients where nephrin and IgG overlap does not exhibit a punctate pattern, detailed investigation using circulating anti-nephrin antibodies and other methods is necessary. Third, this study included only Japanese patients. The incidence of pediatric INS is about three times higher in East Asians, including Japan, than in Europeans and Americans [18–21]. We have also reported that the peak for *NPHS1*, which encodes nephrin, in genome-wide association studies of in pediatric steroid-sensitive nephrotic syndrome is specific to East Asians [22, 23]. This may influence our results, and further examination including genotyping and expanding the study to other ethnicities is necessary. Finally, although our study included various glomerular diseases other than MCD, each condition has a distinct pathogenesis, making it inappropriate to discuss them collectively. Furthermore, even in the acute phase, many cases underwent renal biopsy after some form of therapeutic intervention. However, due to the limited number of patients in each disease category, subgroup analyses were not feasible.

In summary, our comprehensive analysis of kidney specimens has shown that nephrin/IgG co-localization is widely but particularly observed in immune-mediated INS, regardless of the patient’s age and histopathology. Co-localization was observed in parallel with disease activity, and in diseases known to be based on clear genetic factors or other immunological mechanisms, even if IgG staining was positive, co-localization with nephrin was not observed. The exact role of anti-nephrin antibodies in kidney tissue and whether other autoantibodies beyond anti-nephrin antibodies are responsible for INS need to be determined.

## Supplementary Information

Below is the link to the electronic supplementary material.

Supplementary file1 (DOCX 42 KB)

Supplementary file2 (PPTX 353808 KB)

Supplementary file3 (PPTX 44437 KB)

Supplementary file4 (PPTX 29921 KB)

Supplementary file5 (PPTX 771 KB)

## Data Availability

Data from this study can be obtained from the corresponding authors on reasonable request.
