# Supplementary material for: Co-localization of IgG with nephrin in immune-mediated idiopathic nephrotic syndrome
Source: Clin Exp Nephrol. 2025 Aug 6;29(12):1821–8. doi: 10.1007/s10157-025-02741-5 (PMC12660451; doi:10.1007/s10157-025-02741-5)
Supplement: Supplementary file 1 — Supplementary file1 (DOCX 42 KB) [file 10157_2025_2741_MOESM1_ESM.docx]

**Supplementary data**

Co-localization of IgG with nephrin in immune-mediated idiopathic nephrotic syndrome

Yuta Ichikawa^1^, Nana Sakakibara^1^, Shuhei Aoyama^1^, Yuka Kimura^1^, Yuta Inoki^1^, Yu Tanaka^1^, Chika Ueda^1^, Hideaki Kitakado^1^, China Nagano^1^, Tomohiko Yamamura^1^, Shingo Ishimori^1^, Yuko Shima^2^, Hayaki Okamoto^3^, Hideki Fujii^3^, Hironobu Maruyama^4^, Kazumoto Iijima^5, 6^, Kandai Nozu^1^, Tomoko Horinouchi^1^

^1^Department of Pediatrics, Kobe University Graduate School of Medicine, Kobe, Japan.

^2^Department of Pediatrics, Wakayama Medical University, Wakayama City, Japan.

^3^Division of Nephrology and Kidney Center, Kobe University Graduate School of Medicine, Kobe, Japan.

^4^Immuno-Biological Laboratories, Fujioka, Japan.

^5^Hyogo Prefectural Kobe Children's Hospital, Kobe, Japan.

^6^Department of Advanced Pediatric Medicine, Kobe University Graduate School of Medicine, Kobe, Japan.

Corresponding author

Tomoko Horinouchi, M.D., Ph.D.

Department of Pediatrics, Kobe University Graduate School of Medicine

7-5-1 Kusunoki-cho, Chuo, Kobe, Hyogo 6500017, Japan.

Fax: +81-78-382-6099; Tel.: +81-78-382-6090

E-mail: tohori@med.kobe-u.ac.jp

**Supplementary methods**

**Double Immunofluorescence Staining for CD10 and IgG**

Double immunofluorescence staining for CD10 and IgG was performed using frozen kidney biopsy sections. Frozen sections (3 μm thick) were fixed in acetone for 10 minutes and then washed three times with 10% phosphate-buffered saline (PBS). A primary antibody mixture containing anti-CD10 antibody (1:20 dilution, #200103; R&D Systems) and Alexa Fluor 488-labeled anti-IgG antibody (1:100 dilution) was applied, and the sections were incubated for 2 hours at room temperature. After three washes with 10% PBS, sections were incubated for 1 hour with a secondary antibody solution consisting of Goat anti-Mouse IgG (H+L), Highly Cross-Adsorbed Secondary Antibody, Alexa Fluor™ 546 (1:100 dilution; A11030, Invitrogen), mixed with normal goat serum at a 1:20 ratio to achieve a final concentration of 5% for blocking.

Following an additional three washes with 10% PBS, sections were mounted using Vectashield antifade mounting medium with DAPI (H-1200; Vector Laboratories, Newark, CA, USA). The stained sections were examined using a fluorescence microscope equipped with an optical sectioning algorithm (BZ-X810; KEYENCE, Osaka, Japan) to obtain double-immunofluorescence images.

**Table S1**

Clinical features of the 52 enrolled cases

**Table S2**

List of the 68 podocyte-related genes included in the targeted sequencing analysis within a clinically approved gene panel test developed in our laboratory

**Table S3**

Genotype information of five cases for which disease-causing variants were identified

**Fig. S1**

**Results of double-immunofluorescence staining for nephrin and IgG in 52 frozen sections of biopsies from patients with various glomerular diseases**

First column, nephrin staining; second column, IgG staining; third column, merged images for determination of co-localization.

Abbreviations: INS, idiopathic nephrotic syndrome; MCD, minimal change disease; FSGS, focal segmental glomerular sclerosis; NS, nephrotic syndrome; LN, lupus nephritis; MN, membranous nephropathy; IgAN, IgA nephropathy; IgAVN, IgA vasculitis nephritis; MPGN, membranoproliferative glomerulonephritis; GN, glomerulonephritis; MGA, minor glomerular abnormalities

**Fig. S2**

**Nephrin/IgG co-localization in the proximal tubule**

In some cases, nephrin/IgG co-localization was also observed in the proximal tubules. (A) In Case 21, a case of INS in the active phase, IgG and nephrin co-localization was observed both in the glomeruli and in the proximal tubules. (B) In Case 27, a case of INS in remission, although the co-localization in the glomeruli had disappeared, nephrin-bound IgG immune complexes were still detected in the proximal tubules. (C) In Case 21, co-staining of IgG and CD10 demonstrated that IgG is localized within the proximal tubules. (D) In Case 21, although not strictly consecutive, a section containing the same tubules and glomeruli as in (C) showed co-localization of nephrin and IgG.

Abbreviation: INS, idiopathic nephrotic syndrome; FSGS, focal segmental glomerulosclerosis; MCD, minimal change disease

**Fig. S3**

**Classification of nephrin/IgG co-localization-negative pattern**

Merged images of nephrin and IgG staining. In kidney tissues other than in cases of INS, IgG and nephrin were clearly present in different positions. In some cases other than INS, IgG and nephrin were observed along closely parallel lines (A), or IgG and nephrin were observed alternately, in stripes (B, C), but never overlapped. In some INS cases in the active phase, clear deposition of IgG was visible, but no nephrin/IgG co-localization was observed. IgG was clearly deposited on the basement membrane in Case 18 (D) and Case 23 (E). IgG was surrounded by nephrin in Case 12 (F).

Abbreviation: INS, idiopathic nephrotic syndrome; IgAVN, IgA vasculitis nephritis; LN, lupus nephritis; MN, membranous nephropathy

**Fig. S4**

**Nephrin staining negativity in glomeruli of a patient with congenital nephrotic syndrome caused by compound heterozygous *NPHS1* variants**

A congenital nephrotic syndrome patient with compound heterozygous *NPHS1* variants (Case 31) showed no expression of nephrin in the glomeruli, while patients with mild podocyte damage, such as nephrotic syndrome in remission (Case 3) or asymptomatic proteinuria, showed clear expression of nephrin.

**Table S1**

**Clinical features of the 52 enrolled cases**

MCD*: MCD with mesangial proliferation

Abbreviations: SRNS, steroid-resistant nephrotic syndrome; NS, nephrotic syndrome; CyA, cyclosporine A; FRNS, frequent-relapse nephrotic syndrome; MCD, minimal change disease; DMP, diffuse mesangial proliferation; FSGS, focal segmental glomerulosclerosis; LN, lupus nephritis; MN, membranous nephropathy; IgAN, IgA nephropathy; IgAVN, IgA vasculitis nephritis; MPGN, membranoproliferative glomerulonephritis; GN, glomerulonephritis; MGA, minor glomerular abnormalities

**Table S2**

**List of the 68 podocyte-related genes included in the targeted sequencing analysis within a clinically approved gene panel test developed in our laboratory**

| *ACTN4* | *COQ2* | *ITSN1* | *MAGI2* | *PAX2* | *TPRKB* |
| --- | --- | --- | --- | --- | --- |
| *ADCK4* | *COQ6* | *ITSN2* | *MYH9* | *PDSS2* | *TRIM8* |
| *ANKFY1* | *CRB2* | *KANK1* | *MYO1E* | *PLCE1* | *TRPC6* |
| *ANLN* | *CUBN* | *KANK2* | *NPHS1* | *PODXL* | *TTC21B* |
| *ARHGAP24* | *DLC1* | *KANK4* | *NPHS2* | *PRDM15* | *WDR4* |
| *ARHGDIA* | *EMP2* | *KIRREL1* | *NUP85* | *PTPRO* | *WDR73* |
| *AVIL* | *FAT1* | *LAGE3* | *NUP93* | *SCARB2* | *WT1* |
| *CD2AP* | *GAPVD1* | *LAMA5* | *NUP107* | *SGPL1* | *XPO5* |
| *CKD20* | *GON7* | *LAMB2* | *NUP133* | *SMARCAL1* |  |
| *COL4A3* | *INF2* | *LMNA* | *NUP160* | *TNS2* |  |
| *COL4A4* | *ITGA3* | *LMX1B* | *NUP205* | *TBC1D8B* |  |
| *COL4A5* | *ITGB4* | *MAFB* | *OSGEP* | *TP53RK* |  |

**Table S3**

**Genotypes information of 5 cases for which disease-causing variants were identified**

| Patient | Gene | Gene variant | Amino acid variant | ACMG  rating | Evidence of pathogenicity | PMID (if previously reported) |
| --- | --- | --- | --- | --- | --- | --- |
| Case 30 | *NPHS1* (NM_004646.4) | c.2207T>C c.3166+5G>A | p.Val736Ala Exon24 skip | P  LP | PS1, PM1, PM2, PP3, PP5  PS3, PM2, PM3 | #27019444  #Novel |
| Case 31 | *NPHS1* | c.1379G>A c.2515delC | p.Arg460Gln p.Gln839Argfs*8 | LP  P | PS1, PM1, PM2, PP5, BP4  PVS1, PS1, PM2, PP5 | #11317351 #19321760 |
| Case 32 | *SMARCAL1*  (NM_014140.3) | c.1736C>T c.2449C>T | p.Ser579Leu p.Arg817Cys | LP LP | PM1, PM2. PM3, PP3, PP5 PM1, PM2, PM3, PP3 | #11799392 #22998683 |
| Case 48 | *CUBN*  (NM_001081.4) | c.7580_7581del c.6821+3A>G | p.Cys2527^＊^ - | LP  LP | PVS1, PM2  PS1, PM2 | #Novel  #31328266 |
| Case 49 | *COL4A4* (NM_000092.5) | c.2566C>T | p.Gln856^＊^ | P | PVS1. PS1, PM2 | #32734219 |
