# Supplementary material for: Co-localization of IgG with nephrin in immune-mediated idiopathic nephrotic syndrome
Source: Clin Exp Nephrol. 2025 Aug 6;29(12):1821–8. doi: 10.1007/s10157-025-02741-5 (PMC12660451; doi:10.1007/s10157-025-02741-5)
Supplement: Supplementary file 3 — Supplementary file3 (PPTX 44437 KB) [file 10157_2025_2741_MOESM3_ESM.pptx]

## Slide 1
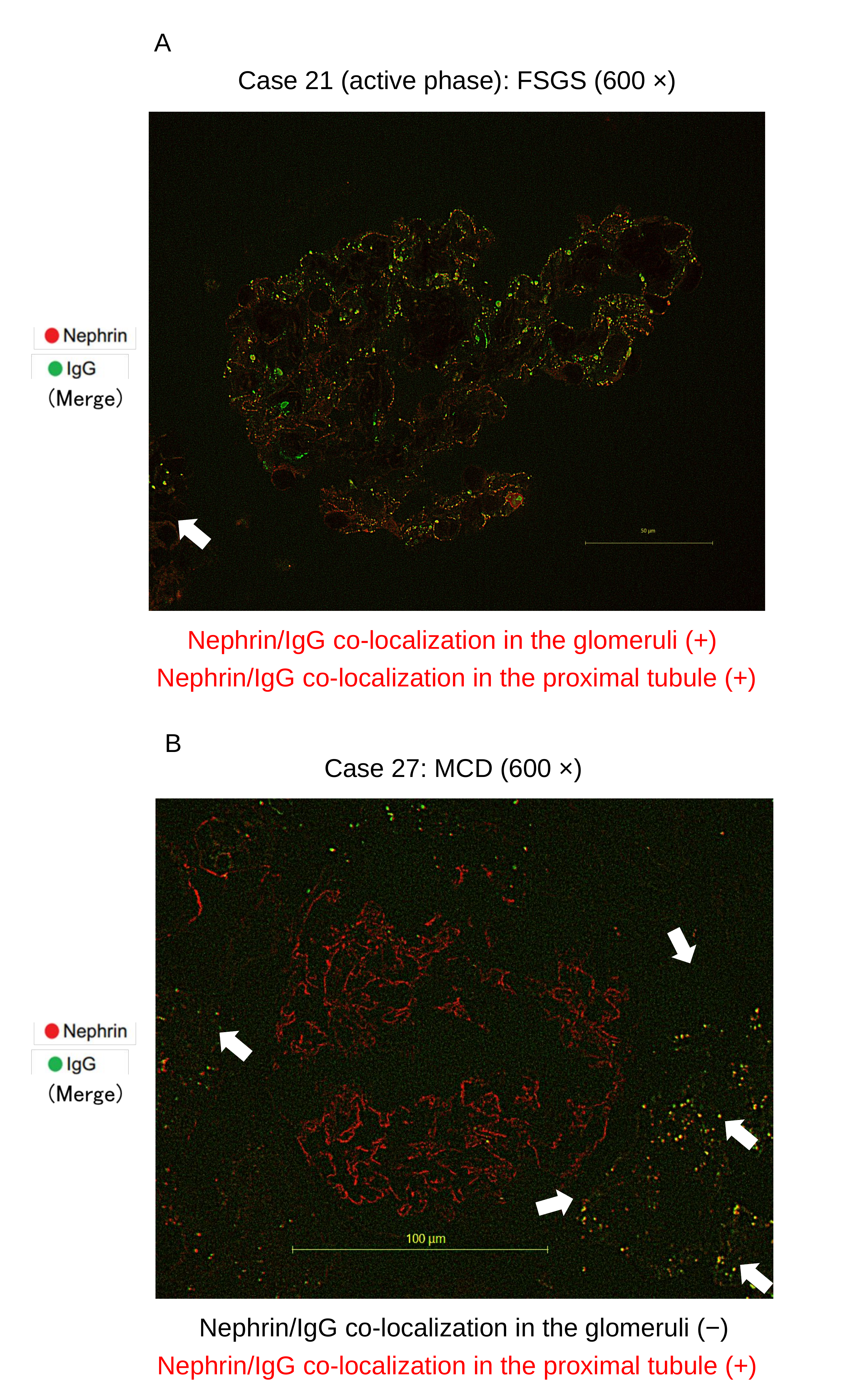

A
Case 21 (active phase): FSGS (600 ×)
Nephrin/IgG co-localization in the glomeruli (+)
Nephrin/IgG co-localization in the proximal tubule (+)
B
Case 27: MCD (600 ×)
Nephrin/IgG co-localization in the glomeruli (−)
Nephrin/IgG co-localization in the proximal tubule (+)

## Slide 2
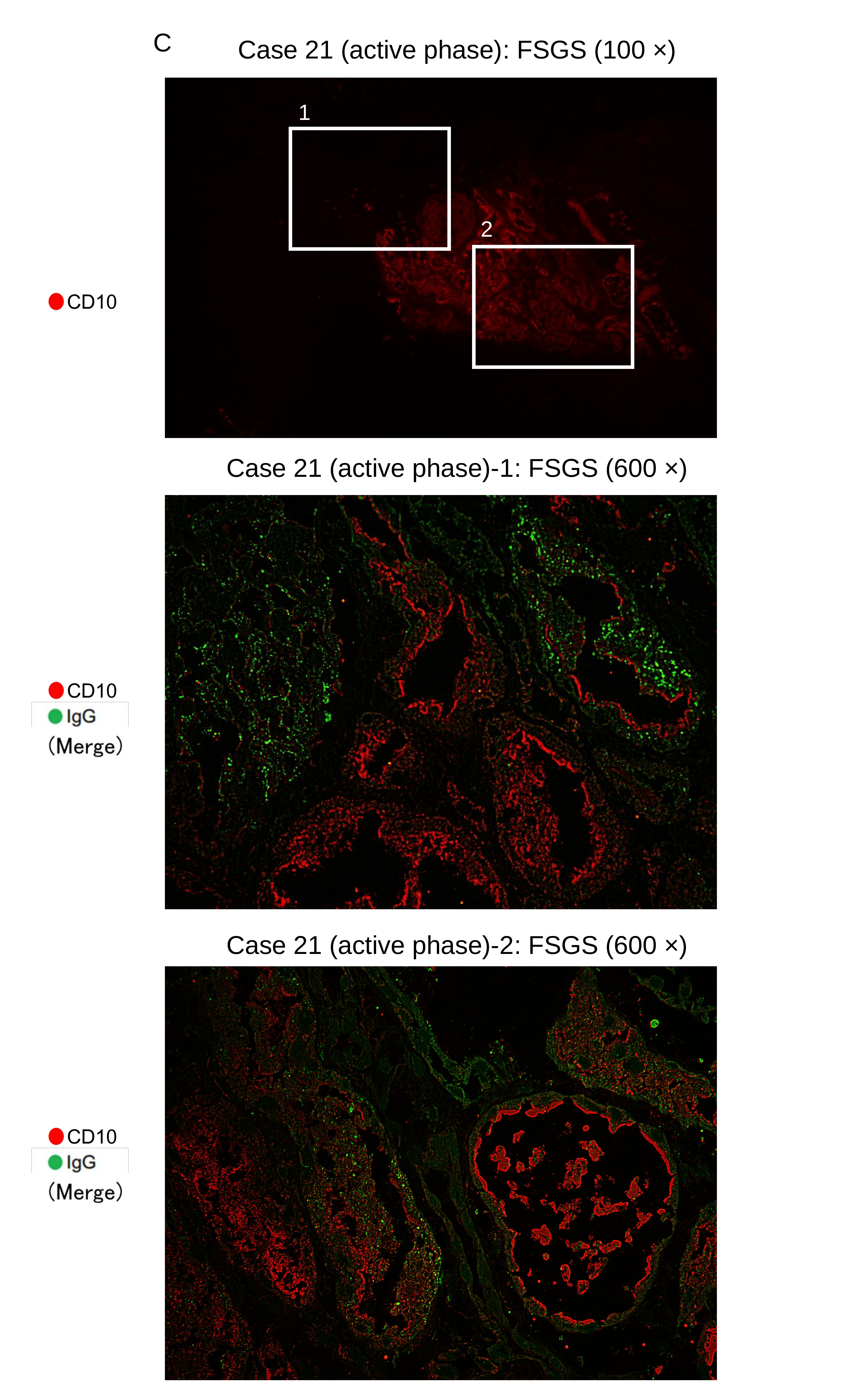

C
Case 21 (active phase): FSGS (100 ×)
1
2
Case 21 (active phase)-1: FSGS (600 ×)
Case 21 (active phase)-2: FSGS (600 ×)

## Slide 3
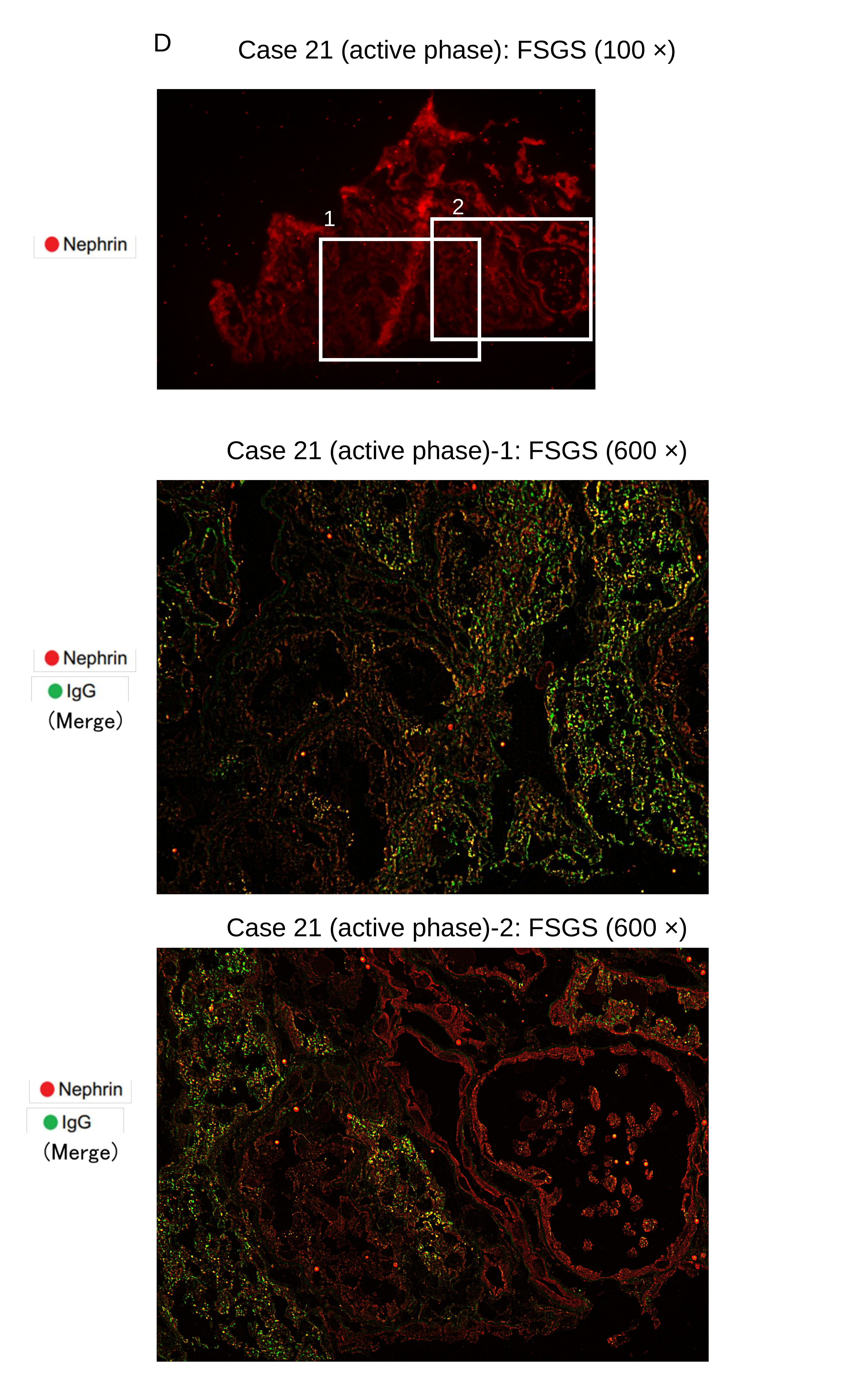

D
Case 21 (active phase): FSGS (100 ×)
2
1
Case 21 (active phase)-1: FSGS (600 ×)
Case 21 (active phase)-2: FSGS (600 ×)
