# Supplementary material for: Co-localization of IgG with nephrin in immune-mediated idiopathic nephrotic syndrome
Source: Clin Exp Nephrol. 2025 Aug 6;29(12):1821–8. doi: 10.1007/s10157-025-02741-5 (PMC12660451; doi:10.1007/s10157-025-02741-5)
Supplement: Supplementary file 4 — Supplementary file4 (PPTX 29921 KB) [file 10157_2025_2741_MOESM4_ESM.pptx]

## Slide 1
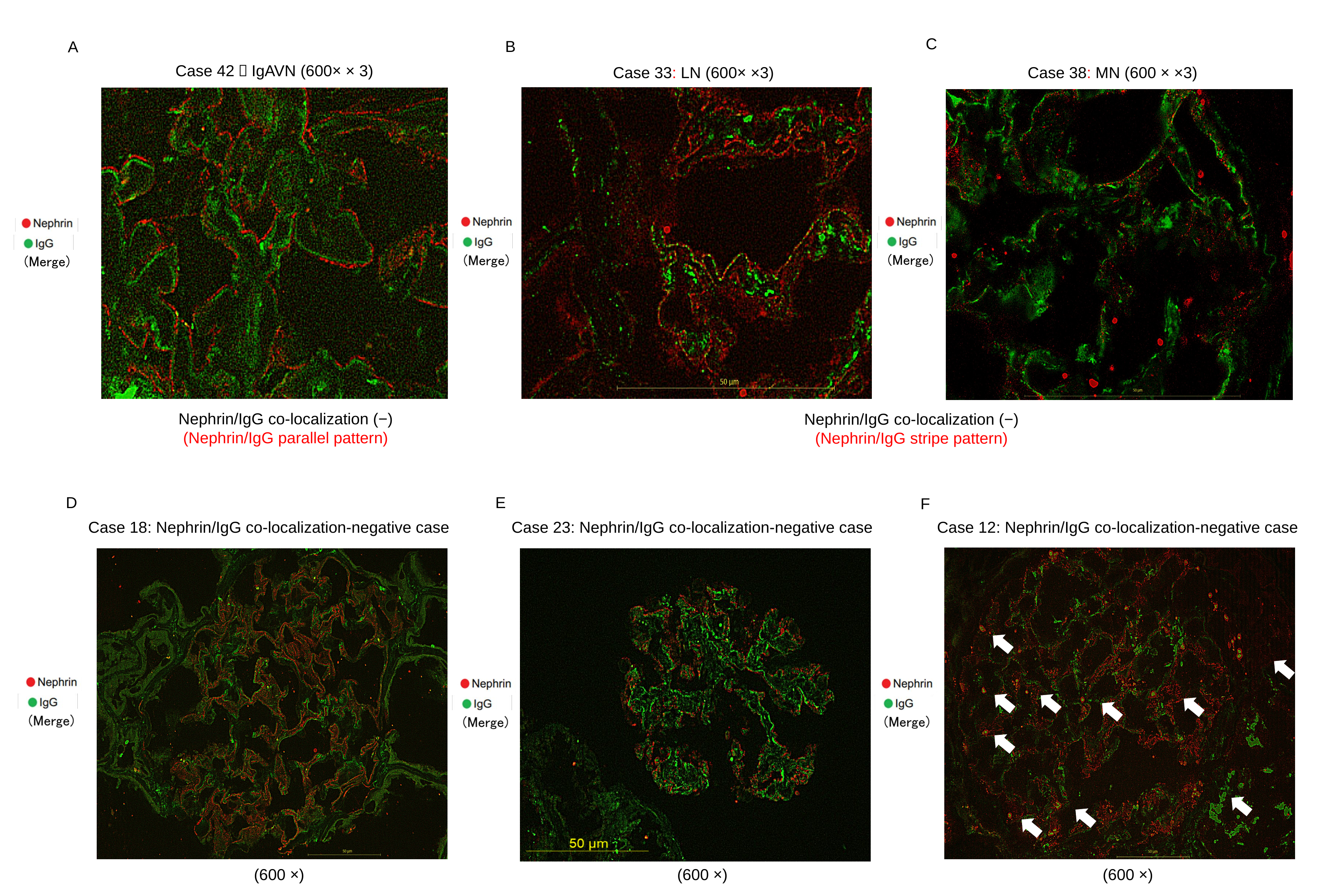

C
B
A
Case 42：IgAVN (600× × 3)
Case 33: LN (600× ×3)
Case 38: MN (600 × ×3)
Nephrin/IgG co-localization (−)
(Nephrin/IgG parallel pattern)
Nephrin/IgG co-localization (−)
(Nephrin/IgG stripe pattern)
D
E
F
Case 23: Nephrin/IgG co-localization-negative case
Case 18: Nephrin/IgG co-localization-negative case
Case 12: Nephrin/IgG co-localization-negative case
 (600 ×)
 (600 ×)
 (600 ×)
