# Supplementary material for: Co-localization of IgG with nephrin in immune-mediated idiopathic nephrotic syndrome
Source: Clin Exp Nephrol. 2025 Aug 6;29(12):1821–8. doi: 10.1007/s10157-025-02741-5 (PMC12660451; doi:10.1007/s10157-025-02741-5)
Supplement: Supplementary file 5 — Supplementary file5 (PPTX 771 KB) [file 10157_2025_2741_MOESM5_ESM.pptx]

## Slide 1
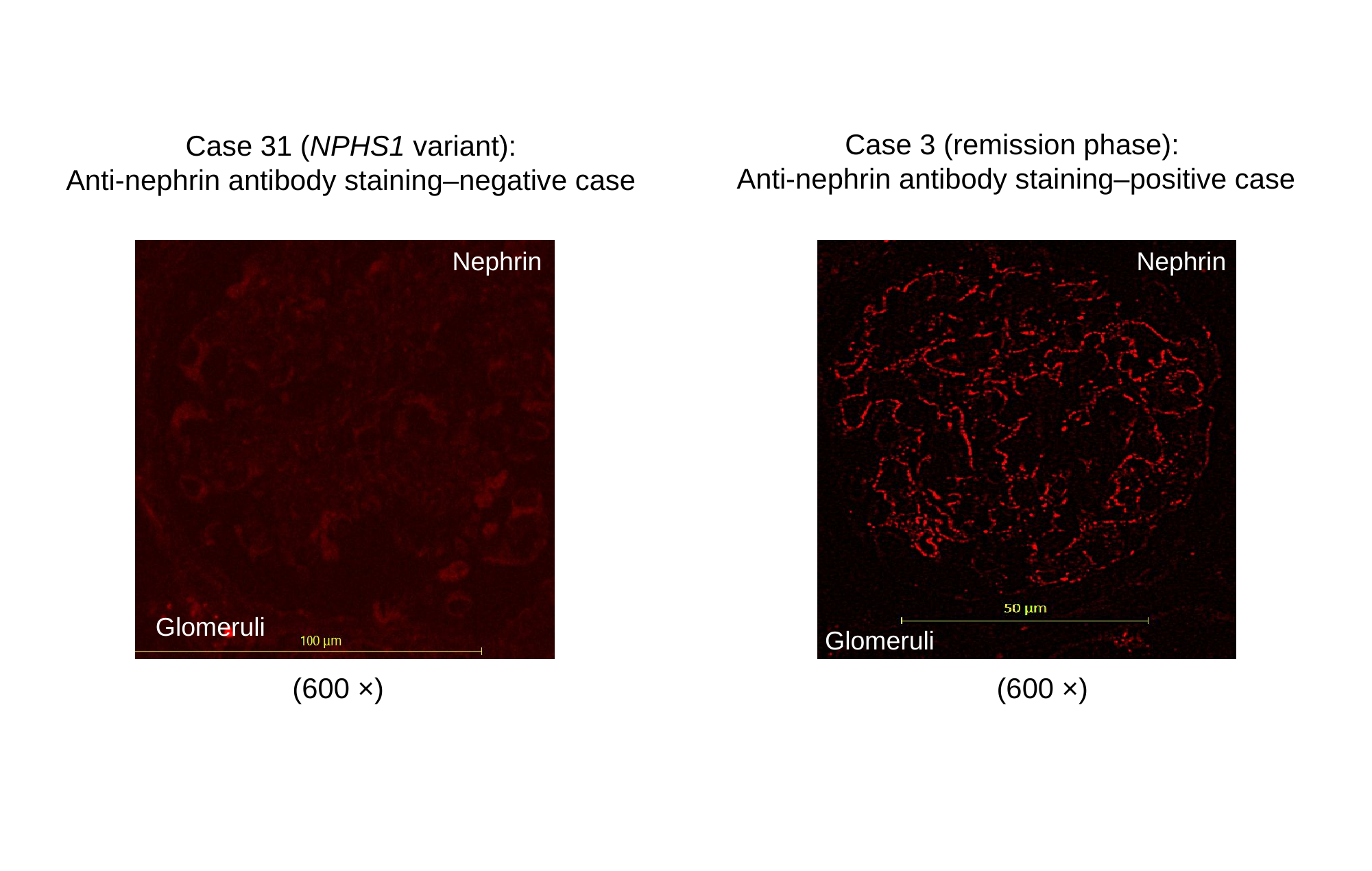

Case 3 (remission phase):
Anti-nephrin antibody staining–positive case
Case 31 (NPHS1 variant):
Anti-nephrin antibody staining–negative case
Nephrin
Nephrin
Glomeruli
Glomeruli
 (600 ×)
 (600 ×)
